# Supplementary material for: Multimodal generative AI for automated pavement condition assessment: Benchmarking model performance
Source: PLoS One. 2026 Feb 12;21(2):e0340380. doi: 10.1371/journal.pone.0340380 (PMC12900301; doi:10.1371/journal.pone.0340380)
Supplement: S1 Table — (DOCX) [file pone.0340380.s001.docx]

| Category | Prompt item | Task Type |
| --- | --- | --- |
| Crack identification | 1.1 Are there any visible cracks on the road surface? | Pavement surface distress and feature identification |
|  | 1.2.1 Do Transverse cracks present? And how many numbers of Transverse cracks are in the image?  1.2.2 Do Longitudinal cracks present? And how many Longitudinal cracks are in the image?  1.2.3 Do Alligator cracks present? And how many Alligator cracks are in the image?  1.2.4 Are there any other types of cracks present on this image | Pavement surface distress and feature identification |
|  | 1.3 If there are visible cracks, are the cracks present with any patterns such as Isolated, Spread across the surface, Along joints/edges? | Damage Pattern Recognition |
|  | 1.4.1 do cracks with Isolated pattern present on the image?  1.4.2 do cracks with Spread across the surface pattern present on the image?  1.4.3 do cracks with Along joints/edges pattern present on  the image?  1.4.4 If there are any other cracks patterns other than Isolated, spread across the surface, along joints/edges present on this image, explain here | Damage Pattern Recognition |
|  | 1.5 Please give an overall description of the cracks issue. | Road condition description and severity evaluation |
| Pothole identification | 2.1 Are there any potholes present on the road surface? | Pavement surface distress and feature identification |
|  | 2.2 Count the number of potholes. | Pavement surface distress and feature identification |
|  | 2.3 Do potholes form a pattern? | Damage Pattern Recognition |
|  | 2.4.1 Do potholes with isolated patterns present in the image?  2.4.2 Do potholes with Spread across the surface pattern present on the image?  2.4.3 Do potholes with Along joints/edges pattern present on the image?  2.4.4 If there are any other patterns other pothole than of Isolated, spread across the surface, along joints/edges present on this image, explain here. | Damage Pattern Recognition |
|  | 2.5 If there are potholes present on the road surface, please give an overall description of the potholes issue. | Road condition description and severity evaluation |
| Severity assessment | 3.1 What is the severity category based on the current road surface condition? Give answers as Excellent, Good, At-risk, Poor, Very Poor. Please also provide the rationale for your categorization. | Road condition description and severity evaluation |
|  | 3.2.1 provide a brief overall road severity assessment.  3.2.2 provide comments on how the road condition affects drivability.  3.2.3 provide comments on how the road conditions affect safety.  3.2.4 provide comments on how the road condition affects future deterioration risks.  3.2.5 provide comments on how the road condition affects other factors not mentioned above. | Road condition description and severity evaluation |
|  | 3.3 What is the estimated pavement condition index value based on the current surface condition? | Road condition description and severity evaluation |
| Environmental and surrounding context | 4.1 Are cracks or potholes more prevalent in specific areas? | Damage Pattern Recognition |
|  | 4.2.1 Are cracks or potholes spread evenly?  4.2.2 Are cracks or potholes located near drainage?  4.2.3 Are cracks or potholes located near intersections?  4.2.4 list any pattern that does not include near drainage, near intersections or spread evenly. | Damage Pattern Recognition |
|  | 4.3 Are cracks or potholes appearing near utility cuts or road construction joints? | Damage Pattern Recognition |
|  | 4.4 Are there any visible signs of poor drainage or standing water that could worsen cracks or potholes? | Pavement surface distress and feature identification |
|  | 4.5 Is there any evidence that this road has been previously repaired? | Pavement surface distress and feature identification |
|  | 4.6.1 Are there any patched areas shown in the image?  4.6.2 Are there any sealed cracks shown in the image?  4.6.3 Are there any signs of resurfacing shown in the image?  4.6.4 List any signs of fixed road that do not include patched areas, resurfacing, or sealed cracks. | Pavement surface distress and feature identification |
| Future repairment | 5.1 How soon should this road be repaired? Short-term (should be repaired within one year); Long-term (should be repaired within one to three years); No repair needed (no repairs needed for at least three years). | Repair forecasting |
