# Supplementary material for: Multimodal generative AI for automated pavement condition assessment: Benchmarking model performance
Source: PLoS One. 2026 Feb 12;21(2):e0340380. doi: 10.1371/journal.pone.0340380 (PMC12900301; doi:10.1371/journal.pone.0340380)
Supplement: S2 Table — (DOCX) [file pone.0340380.s002.docx]

| Street Coordination | Condition Evaluation |
| --- | --- |
| 37.73925375, -122.4770299 | Recommended by the City of San Francisco (conducted in September 2023):   - PCI Score: 80 - PCI Condition Level: Very good   Frequent MLLM-Based Condition Evaluation:   - PCI Condition Level: At-risk |
| 37.78225119, -122.4496156 | Recommended by the City of San Francisco (conducted in September 2023):   - PCI Score: 40 - PCI Condition Level: Poor   Frequent MLLM-Based Condition Evaluation:   - PCI Condition Level: Good |
| 37.8014441, -122.4045067 | Recommended by the City of San Francisco (conducted in March 2012):   - PCI Score: 37 - PCI Condition Level: Poor   Frequent MLLM-Based Condition Evaluation:   - PCI Condition Level: Good/At-risk |
