# Supplementary material for: Multimodal generative AI for automated pavement condition assessment: Benchmarking model performance
Source: PLoS One. 2026 Feb 12;21(2):e0340380. doi: 10.1371/journal.pone.0340380 (PMC12900301; doi:10.1371/journal.pone.0340380)
Supplement: S3 Table — (DOCX) [file pone.0340380.s003.docx]

| Street Coordination | Condition Description |
| --- | --- |
| 37.72858705, -122.3788582 | Recommended by the City of San Francisco (conducted in September 2024):   - PCI Score: 80 - PCI Condition Level: Very Good - Actual Repair History: No repairs have been made for at least three years.   Most Common MLLM Repair Recommendation:   - Short-term (should be repaired within one year) |
| 37.80351658, -122.4224456 | Recommended by the City of San Francisco (conducted in October 2016):   - PCI Score: 30 - PCI Condition Level: Poor - Actual Repair History: No repairs have been made for at least three years.   Most Common MLLM Repair Recommendation:   - Short-term (should be repaired within one year) |
