# Supplementary material for: Multimodal generative AI for automated pavement condition assessment: Benchmarking model performance
Source: PLoS One. 2026 Feb 12;21(2):e0340380. doi: 10.1371/journal.pone.0340380 (PMC12900301; doi:10.1371/journal.pone.0340380)
Supplement: Attachment — Submitted filename: Response to Reviewers.docx [file pone.0340380.s005.docx]

**Manuscript PONE-D-25-46997**

**Response to Editor and Reviewers**

Additional Editor Comments (if provided):

Please ensure to address the reviewers' comments thoroughly. Additionally, please expand the literature review of recent papers on this topic to discuss gaps in existing studies and why those gaps are significant. There are many papers on similar or relevant topics (i.e., LLM + Street-view images) published in geography and urban planning journals, which have not been extensively discussed in this manuscript.

**Author response:** Thank you for the valuable feedback. We have expanded the literature review to clarify the research gap and explain its significance, thereby highlighting the study’s contribution.

- Added references 26, 60–69 to highlight that existing machine learning approaches for surface road condition assessment are typically designed to perform a single task per model, whereas MLLMs can handle multiple tasks within a unified framework.
- Added references 58–59 to indicate that MLLMs can achieve high performance with substantially less training data compared to traditional machine learning approaches.

We also have expanded the literature review to include recent studies on similar and relevant topics, particularly those integrating large language models and street-view imagery in urban analysis.

- Added references 29–31 to support the statement that researchers have increasingly leveraged MLLMs in urban studies by applying street-view imagery for a range of analytical tasks.
- Added references 31 and 73–80 to provide further detail on how MLLMs have been used for urban analytics through street-view images.

**Reviewers' Comments to the Authors: Reviewer**

**Reviewer #1**:

This paper proposes using large language models (LLMs) to audit pavement conditions. I think many technical details need to be described and considered more carefully.
Abstract
1. Page 1: The abstract starts by saying LLMs show promise in automated assessment of many things, but automated pavement condition assessment is unexplored. I don’t think this is a strong motivation (there are countless unexplored applications of LLMs). Instead, the authors should start with current challenges in pavement condition assessment and then explain how LLMs could help address these challenges.

**Author response:** Thank you very much for pointing this out. We agree that the abstract should begin with the challenges of current pavement condition assessment rather than focusing on unexplored applications of large language models. We have revised the abstract to highlight the limitations of existing approaches, such as high costs, labor-intensive processes, and limited scalability, before introducing how multimodal large language models (MLLMs) can help address these challenges.

The revised text now reads at the beginning of the abstract as follows:

“Accurate and efficient pavement condition assessment is essential for maintaining roadway safety and optimizing maintenance investments. However, conventional assessment methods such as manual visual inspections and specialized sensing equipment are often time-consuming, expensive, and difficult to scale across large networks.”

Introduction
2. Page 2 (lines 36-39): Proper references should be cited for existing work on “structural evaluation” and “surface-level evaluation.” The authors should also specify which physical characteristics these studies focus on, with a few examples.

**Author response:** Thank you for the helpful suggestion. We have added references to previous studies that discuss both structural and surface-level evaluation methods and clarified the physical characteristics examined in these assessments. The revised text is provided below.

“Road condition assessment methods range from structural evaluations that measure subsurface integrity using techniques such as falling weight deflectometer testing (7, 8) and ground-penetrating radar (9, 10), to surface-level inspections, which analyze visible pavement conditions such as cracking, rutting, raveling, and potholes (11-14).”

3. Page 3 (lines 46-56): Several claims here seem factually inaccurate. First, when you say existing deep learning models require “high-quality, structured image data,” do you mean training data? Even for LLMs, you still need high-quality image data to support detection and decision-making. Second, while deep learning models need human oversight, so do LLMs, which can hallucinate and require human review (so they can’t truly autonomously make decisions). Perhaps the authors mean that a single LLM could potentially handle not just detection but also evaluation of severity and maintenance timing, tasks that would require multiple deep learning models.

**Author response:** To address your first point, we agree that our wording was unclear. We intended to refer to training data when stating that existing deep learning models require “high-quality, structured image data.” Traditional deep learning methods rely on large, labeled datasets for training before they can generate accurate predictions. In contrast, MLLMs can process new images without task-specific training. However, their performance still depends on the quality of the image inputs and may require fine-tuning once benchmark data becomes available.

Regarding your second point, we fully agree that both deep learning models and MLLMs require human oversight, as MLLMs can hallucinate or misinterpret visual information. Our intention was to emphasize that MLLMs have the potential to handle multiple subtasks such as defect detection, severity evaluation, and maintenance prioritization within a single model, whereas traditional deep learning approaches typically require separate models for each task.

For better clarity, we added supporting references and revised the paragraph as follows:

“Although MLLMs have not yet reached the point of full automation, they are more adaptable than traditional machine learning models, particularly in low-resource environments, and can operate effectively with smaller or less-structured datasets (56).”

“Developing these models requires large volumes of high-quality, accurately labeled training data (23, 24). Acquiring such data is often challenging and time-consuming due to the need for extensive manual annotation (25). In addition, most models are designed for a single, task-specific function (26). Since road surface assessment involves multiple stages, including defect detection, severity evaluation, and maintenance prioritization, separate models are typically required to address each component of the workflow.”

Methods
4. Page 7: First, what are the 39 prompts used? These should be spelled out either here or in an appendix, as it is currently unclear what the authors are asking the LLMs to do. Second, how are the model parameters set, such as temperature, top-k, etc.? Third, for each LLM, is it only prompted once per street view image? Typically, to enhance robustness in LLM-assisted assessment, each LLM should be prompted multiple times per image, because responses can vary due to inherent randomness.

**Author response:** We sincerely appreciate the reviewer’s thoughtful comments and suggestions. We have revised the manuscript to clarify the missing information regarding prompt design, model configuration, and the prompting procedure.

- **Prompts:** The complete list of 39 prompts used in this study has been added to S1 Table. These prompts were carefully designed to evaluate four key aspects of road condition assessment: surface feature identification, pattern recognition, severity evaluation, and repair forecasting.

The revised text now reads as follows:

“A total of 39 unique prompts were developed, as detailed in S1 Table.”

- **Model parameters:** All models were configured with a temperature of 0.3 and top-p of 1.0, while other parameters remained at their default settings to ensure consistency across platforms.
- **Prompting procedure:** Each image was prompted once. This approach was chosen because the 39 prompts were designed primarily as close-ended questions with well-defined terms and clearly worded instructions (for example, yes/no, multiple choice, or numeric rating), which limit open-ended variability in model outputs. The text outputs across models also showed high internal consistency, particularly among proprietary models such as Gemini 2.5 Pro, GPT-4o, and OpenAI o1. Furthermore, preliminary testing confirmed that repeated prompting produced nearly identical results, indicating that a single prompt per image was sufficient for reliable analysis under these controlled settings.

For improved clarity, the revised text in the main manuscript now reads as follows:

“This study evaluated the performance of seven MLLMs: Gemini 2.5 Pro, OpenAI o1, GPT-4o, Gemma 3, Llama 3.2, LLaVA v1.6 Mistral, and LLaVA v1.6 Vicuna. Figure 1 shows the general workflow of the MLLM evaluation process. These models were tested across five performance dimensions to assess their ability to complete four categories of road condition assessment tasks of varying complexity. Each image was prompted once, as the 39 prompts were designed primarily as close-ended questions with well-defined terms and clear worded instructions (e.g., yes/no, multiple choice, or numerical rating), which limit open-ended variability in model outputs. All models were configured with a temperature of 0.3 and a top-p of 1.0, while other parameters were kept at their default settings to ensure consistency across platforms. All evaluations were conducted on a high-performance computing cluster equipped with NVIDIA V100 GPUs (32 GB memory) and Intel Xeon Gold 6148 CPUs (2.40 GHz).”

5. Page 8: More details are needed about the manual annotation process. For example, where were the annotators recruited? Provide a detailed list of labels used for surface features and spatial distribution patterns, not just examples. The examples given here could instead go in the Introduction (page 3, lines 57–63), because terms like “spatial distribution pattern” are unclear until this point.

**Author response:** Thank you for the helpful suggestion. We have expanded the section describing the manual annotation process to clarify annotator recruitment and provide a detailed list of annotation labels. The revised text reads as follows:

“In addition, manual annotations were conducted by members of the research team to support the evaluation of the models’ identification capabilities. Each image was independently reviewed by annotators, who answered a series of structured questions corresponding to the model prompts. A total of 29 questions were answered for each image. These questions focused on the presence and type of pavement defects, their spatial distribution, and relevant environmental or contextual conditions. Specifically, annotators assessed (1) surface features, such as whether cracks or potholes were present, and if so, the general type of cracking (transverse, longitudinal, or alligator); (2) spatial distribution patterns, indicating whether defects appeared isolated, spread across the pavement surface, or concentrated along joints, edges, or drainage areas; and (3) environmental and contextual conditions, including visible intersections, drainage features, patched or sealed areas, signs of resurfacing, and evidence of poor drainage or standing water that could exacerbate pavement deterioration. In cases where annotators disagreed, the discrepancies were discussed collectively until a consensus was reached.”

In addition, we have expanded the Introduction (page 3, lines 57–63) to clarify the term like “spatial distribution pattern,” as suggested. The revised text reads as follows:

“To contribute to this emerging area, this study examines the potential of MLLMs assessing road surface conditions based on the physical state of pavement. The evaluation focuses on four key tasks relevant to pavement management workflows: (1) surface distress and feature identification (e.g., cracks, potholes, and patches), (2) spatial pattern recognition (e.g., whether defects are isolated, uniformly distributed, or concentrated along edges or drainage areas), (3) severity evaluation (estimating the extent or seriousness of observed defects), and (4) maintenance interval estimation (inferring the timing or urgency of potential maintenance needs). These tasks were selected for their direct relevance to operational decision-making within pavement condition assessment frameworks.”

6. Page 11: How do you distinguish between response correctness and hallucination in practice? Both result in a mismatch between human-annotated data and LLM responses.

**Author response:** Thank you for this insightful question. We have clarified in the manuscript how response correctness and hallucination are conceptually and analytically distinguished. The clarification has been added to the section ***Assessment dimension***. Specifically, response correctness measures whether the model’s output aligns with the ground-truth annotations based on factual accuracy or appropriate classification, while hallucination refers to fabricated or unsupported content that does not appear in the input image or prompt context. Although both may result in mismatches with human annotations, they differ in their source and nature: incorrect responses reflect misclassification or misunderstanding of visual cues, whereas hallucinations involve the generation of non-existent or irrelevant features.

We also expanded the explanation to emphasize that correctness and hallucination are evaluated from different analytical perspectives. Correctness represents a quantitative, data-driven assessment of model performance, while hallucination pertains to reasoning and interpretive behavior. Understanding hallucination helps explain why incorrect or fabricated content is produced, which in turn informs strategies to improve model correctness.

The revised text now reads as follows:

“Response correctness was used to evaluate the factual accuracy of model outputs by comparing them against benchmark references, including PCI scores, maintenance interval estimates, and manually annotated surface features. For classification-type prompts with predefined label sets, standard metrics such as accuracy, precision, recall, and F1 score were calculated to quantify the alignment between model predictions and ground-truth annotations.”

“Multimodal errors were examined to identify failures in the model’s ability to process, integrate, or reason across visual and textual information within street-view imagery. This analysis focused on the model’s reasoning and interpretive behavior to explore why incorrect or fabricated content was generated. Hallucinations were treated as a specific subtype of multimodal error and were defined as responses that introduced fabricated, unsupported, or irrelevant content not grounded in the input image or prompt. A response was classified as a hallucination when the described feature or condition could not be verified in the corresponding image.”

7. Echoing my earlier points, the authors should be explicit about what they instruct the LLMs to do, what outputs are expected, and what is contained in the human-annotated data. Without this clarity, it is difficult to understand how performance evaluation is conducted. How do we know whether the human-annotated results and LLM responses are comparable?

**Author response:** Thank you very much for this helpful comment. We have expanded the manuscript to clarify (1) what the LLMs were instructed to do, (2) what types of outputs were expected, and (3) what the human-annotated data contained. Specifically:

- Instructions to LLMs: The complete set of 39 prompts used for model evaluation has been added to Appendix A. Each image was prompted once with these 39 questions, and the output consisted of a text-based response for each question.
- Expected outputs: Revised text in the section ***Multimodal input***:

“Each image was prompted once, as the 39 prompts were designed primarily as close-ended questions with well-defined terms and clearly worded instructions (e.g., yes/no, multiple choice, or numerical rating), which limit open-ended variability in model outputs. The output from each model consisted of text-based responses generated in direct answer to the prompt questions, which were then compared with human-annotated responses to evaluate correctness, consistency, and reasoning quality. All models were configured with a temperature of 0.3 and a top-p of 1.0, while other parameters were kept at their default settings to ensure consistency across platforms.

- Human-annotated data: Revised text in the section ***Benchmark data***:

“Manual annotations were conducted by members of the research team to support 6the evaluation of the models’ identification capabilities. Each image was independently reviewed by annotators who answered a series of structured questions corresponding to the model prompts. These questions focused on the presence and type of pavement defects, their spatial distribution, and relevant environmental or contextual conditions. Specifically, annotators assessed (1) surface features, such as whether cracks or potholes were present and, if so, the general type of cracking (transverse, longitudinal, or alligator); (2) spatial distribution patterns, indicating whether defects appeared isolated, spread across the pavement surface, or concentrated along joints, edges, or drainage areas; and (3) environmental and contextual conditions, including visible intersections, drainage features, patched or sealed areas, signs of resurfacing, and evidence of poor drainage or standing water that could exacerbate pavement deterioration.”

**Reviewer #2**: The manuscript titled “Multimodal Generative AI for Automated Pavement Condition Assessment: Benchmarking Model Performance” addresses an important and timely problem by exploring the application of multimodal generative AI to pavement condition assessment. The study provides a clear comparative evaluation of multiple proprietary and open-source models and presents results that highlight relative performance differences across tasks and dimensions. Here are my comments.

1. While the study demonstrates that multimodal generative AI models can produce pavement maintenance recommendations, one important limitation not addressed in the manuscript is the lack of explainability of model outputs. For infrastructure management such as pavement condition assessment, where decisions directly affect safety, costs, and policy priorities, explainability is critical. Without interpretable reasoning or transparent links between input features (e.g., crack severity, traffic data) and recommendations, practitioners may be reluctant to trust or adopt such AI systems. The authors should discuss how LLMs address this.

**Author response:** Thank you for highlighting this important point. We have added a new paragraph in the Limitations section (the section ***The potential and current limitations of existing MLLMs in road surface condition assessment***) to address the issue of explainability. The added text reads as follows:

“Finally, an additional limitation lies in the explainability of MLLM outputs. Although the models can generate decisions related to surface road condition assessment, their reasoning processes remain opaque. For instance, when asked to provide a PCI score based on an image, the models typically return only a numerical value without clarifying how it was derived. While MLLMs can produce descriptive explanations, these are not always grounded in verifiable causal relationships and may reflect post-hoc rationalizations rather than genuine interpretive reasoning. Prompting models to explicitly state the rationale behind their decisions could improve transparency and facilitate more trustworthy applications in pavement assessment.”

2. As one of the limitations of manual inspections, the paper noted that manual inspections are subject to observer variability (ln 40-41). This is also applicable in utilizing generative AI too. Many models generate probabilistic outputs, meaning repeated runs may not always yield identical results unless parameters are fixed. This needs to be acknowledged in this study.

**Author response:** Thank you for pointing this out. We have strengthened the discussion and added a new paragraph in the section ***The potential and current limitations of existing MLLMs in road surface condition assessment*** to address this limitation. The added text reads as follows:

“Although generative AI can reduce reliance on manual inspections by automating visual assessments, human oversight remains necessary to validate cases and ensure alignment with engineering standards. In addition, generative models may produce probabilistic outputs, meaning that repeated runs do not necessarily yield identical results unless generation parameters are fixed. This stochastic variability parallels observer inconsistency in manual inspections and can introduce uncertainty into the evaluation process. Standardizing model parameters and employing well-structured prompts can mitigate this randomness, thereby enhancing the reproducibility and reliability of MLLM outputs.”

3. For the 39 prompts used in this study, are there any domain expertise involved in their design? Given that prompt clarity and completeness strongly affect model outputs, it will benefit this paper to clarify whether experts in pavement management were consulted, and what criteria guided prompt construction.

**Author response:** Thank you for this helpful comment. The 39 prompts were designed around four core tasks that are widely recognized in surface road condition assessment: (1) surface distress and feature identification, (2) spatial pattern recognition, (3) severity evaluation, and (4) maintenance interval estimation. These tasks guided the structure of the prompt set to ensure that the evaluation aligned with established practices in pavement management. Although no formal consultation with external experts was conducted, the prompts were reviewed internally by researchers with expertise in transportation and pavement management to ensure technical relevance and clarity. We have also expanded the literature review to demonstrate that these four tasks represent standard procedures in pavement condition assessment. References 22, 34, 36, and 81–87 have been added, as shown in the revised text below:

“This study evaluates the performance of MLLMs across four tasks relevant to pavement condition assessment. The selection of tasks reflects common practices in surface road condition assessment: (1) surface distress and feature identification (22, 36, 81, 82), (2) spatial pattern recognition (i.e., crack positioning) (83, 84), (3) severity evaluation (34, 85, 86), and (4) maintenance interval estimation (34, 87)”

4. Response rate was used as one of the assessment dimensions. The paper does not clearly explain how response rate was measured in practice. Was it calculated manually?

**Author response:** Thank you for this helpful comment. We have expanded the ***Methods*** section to clarify how the response rate was measured. The revised text now reads:

“Response rate was used to assess model responsiveness. It was defined as the proportion of prompts for which the model generated a valid and interpretable output. A higher response rate indicates that the model can engage with a wide range of task instructions, while a lower rate suggests difficulty in interpreting or addressing certain prompt types. In this study, the response rate was calculated programmatically as the percentage of images that yielded a valid response for each question. Response rates were first computed for each of the 39 prompts for each model and then aggregated to represent overall responsiveness at the model level.”

**Reviewer #3:** The manuscript presents a timely contribution to the growing field of using MLLMs for imagery analysis. The integration of GenAI into road surface monitoring is novel, and the systematic evaluation across different proprietary and open-source models provides useful insights. The study is well motivated and relevant to both urban/transportation planning as well as AI research. But overall, the manuscript is not yet ready for publication. With clearer descriptions, stronger methodology, and better figure clarity, it could become a solid contribution to AI in transportation infrastructure. MAJOR REVISIONS REQUIRED.

Abstract:
1. 23-25: Clear but the claim that GPT 4o provides the most favorable balance between accuracy and cost should be carefully stated.

**Author response:** Thank you for pointing this out. We have revised the sentence to be more cautiously stated:

“Among the evaluated models, we recommend GPT-4o as the preferred option, as it balances responsiveness, accuracy, and computational cost.

Introduction:
2. Highlights the importance of pavement condition assessment and the potential of generative AI, but it does not sufficiently discuss the study in relation to existing work.

**Author response:** Thank you for this valuable comment. We have expanded the introduction to better connect pavement condition assessment and the potential of generative AI to our study. The following paragraph has been added to clarify how our work builds upon and extends existing research:

“However, the application of MLLMs using street-view remain for surface-level road condition assessment remains largely unexplored. Pavement evaluation typically involves detecting surface distress, classifying severity, and recommending maintenance actions, which have traditionally required multiple specialized models. With their multimodal understanding and generative capabilities, MLLMs have the potential to integrate these tasks within a single framework and streamline the assessment process. Before MLLMs can be reliably adopted for pavement evaluation using street-view images, it is important to benchmark their current capabilities, assess their performance across key road condition assessment tasks, and identify areas that require further refinement and adaptation.”

3. prior work in deep learning and computer vision for crack detection and PCI estimation mentioned but does not reference or contrast with these efforts in detail

**Author response:** Thank you for pointing this out. We have expanded the introduction to provide a more detailed discussion of prior work in deep learning and computer vision for pavement assessment and to clarify how our study builds upon these efforts. The revised text now reads:

“Conventional road surface assessments have traditionally relied on manual inspection techniques, which are often time-consuming, labor-intensive, and subject to observe variability. In response, recent developments in computer vision and artificial intelligence have enabled the adoption of automated, image-based methods (15-17). Deep learning (15, 18) and other machine learning techniques (19, 20) are widely applied in pavement condition assessment to enhance accuracy, consistency, and operational efficiency (19, 21, 22). Developing these models requires large volumes of high-quality, accurately labeled training data (23, 24). Acquiring such data is often challenging and time-consuming due to the need for extensive manual annotation (25). In addition, most models are designed for a single, task-specific function (26). Since road surface assessment involves multiple stages, including defect detection, severity evaluation, and maintenance prioritization, separate models are typically required to address each component of the workflow.

More recently, advances in generative artificial intelligence (AI), particularly multimodal models, have gained attention for their potential to move beyond traditional support functions and autonomously generate task-relevant outputs with minimal contextual input. Unlike conventional machine learning models that require extensive training datasets and pre-defined rules, generative AI models can respond adaptively to a range of inputs and generate task-relevant outputs with limited contextual information (27, 28). Leveraging these capabilities, recent studies have begun to apply MLLMs in urban research, particularly using street-view imagery to analyze built environments, urban form, and neighborhood conditions (29-31).

However, the application of MLLMs using street-view remain for surface-level road condition assessment remains largely unexplored. Pavement evaluation typically involves detecting surface distress, classifying severity, and recommending maintenance actions, which have traditionally required multiple specialized models. With their multimodal understanding and generative capabilities, MLLMs have the potential to integrate these tasks within a single framework and streamline the assessment process. Before MLLMs can be reliably adopted for pavement evaluation using street-view images, it is necessary to benchmark their current capabilities, assess their performance across key road condition assessment tasks, and determine the degree of fine-tuning and adaptation required for reliable application in pavement condition assessment.”

4. novelty of benchmarking multimodal generative models is not sharply presented
**Author response:** Thank you for pointing this out. We have clarified the motivation and significance of benchmarking MLLMs to better highlight the novelty of our study. Specifically, we added the following sentence:

“Before MLLMs can be reliably adopted for pavement evaluation using street-view images, it is necessary to benchmark their current capabilities, assess their performance across key road condition assessment tasks, and determine the degree of fine-tuning and adaptation required for reliable application in pavement condition assessment.”

5. The introduction would benefit from more explicitly stating how this study differs from prior research
**Author response:** Thank you for this helpful suggestion. We have revised the introduction to clarify how this study differs from previous work and to highlight its novel contributions. The following paragraph has been added:

“Beyond establishing baseline benchmarks, this study expands prior work by comparing the performance, reliability, and computational efficiency of multiple MLLMs. Although earlier studies have explored the use of individual MLLMs with street-view imagery, they primarily focused on demonstrating the potential of a single model. To address this gap, the present study evaluates the capability of multiple MLLMs for surface-level road condition assessment and examines performance variations across key tasks. Both proprietary and open-source models are included to capture a comprehensive view of current technological capacity, as they differ in architecture, training data, and accessibility. Evaluating both types is important because it enables an assessment of whether open-source alternatives can achieve performance comparable to commercial systems, which has implications for cost-effectiveness and the feasibility of large-scale adoption by public agencies and researchers. The analysis also accounts for computational cost, providing insight into the trade-offs between performance and efficiency.

Methods
6. dataset size is small: needs justification

**Author response:** Thank you for this helpful comment. We have clarified the reasons for the small dataset in both the response and the manuscript. The dataset is relatively small because real-world Pavement Condition Index (PCI) data are scarce and currently available only from the City of San Francisco. The San Francisco dataset provides PCI values and geographic coordinates but does not include street-level imagery, so corresponding Google Street View images were retrieved separately to supplement the analysis. In addition, many road segments in San Francisco have PCI scores of 100, leaving few samples with visible surface defects for evaluation. Finally, only segments where PCI data and Google Street View images were collected within the same one-year period were included to ensure temporal consistency between the two data sources.

We have incorporated these details into the section on ***multimodal input*** in the revised manuscript:

“The input to the MLLMs consisted of street-level road images and textual prompts designed to reflect real-world road condition assessment tasks. The images were sourced from Google Street View and selected based on PCI records, as detailed in the section on ***benchmark data***. To ensure temporal alignment between pavement condition data and Street View imagery, only images captured within one year of a PCI assessment were included. PCI records prior to 2014 were excluded due to the lack of reliably dated imagery. The dataset size is relatively small primarily because real-world PCI data are scarce and currently available only from the City of San Francisco. The available dataset contains PCI values and geographic coordinates but does not include street-level imagery, requiring the retrieval of corresponding Google Street View images for each road segment. Furthermore, many segments in San Francisco have perfect PCI scores (i.e., PCI = 100), resulting in limited representation of visible pavement defects. To mitigate potential sampling bias, a random subset of those records was selected to balance the distribution of pavement conditions. This process resulted in a dataset of 261 road segment images.”

7. 157–160: “ground truth” labels for maintenance interval based on temporal comparisons of Google Street View imagery, which may not reliably capture the actual timing or type of repairs. This introduces potential uncertainty in the benchmark labels

**Author response:** Thank you very much for this thoughtful comment. We agree that using temporal comparisons of Google Street View imagery may introduce some uncertainty in identifying the timing and type of pavement repairs. However, no publicly available dataset currently contains both PCI data and detailed records of actual repair timing or repair types. To ensure consistency, we aligned the identification of maintenance intervals as closely as possible with the PCI dataset. Instead of determining exact repair dates, we categorized maintenance timing into broader and more flexible intervals: within one year, between one and three years, and more than three years. Our research team also made careful efforts to verify repair types by matching imagery from the same locations to maintain spatial consistency. We have added the following clarification to the manuscript to acknowledge this limitation:

“Road maintenance intervals were estimated by reviewing sequential Google Street View images for visible signs of surface repair. Segments showing repairs within one year of the initial image were labeled as short-term maintenance. Repairs that occurred between one and three years later were labeled as long-term. Segments with no visible repairs within three years were classified as having no maintenance. These labels provide a basis for evaluating the models’ ability to estimate repair urgency. While the use of Google Street View imagery may introduce some uncertainty in identifying the exact timing or type of repair, this approach remains a practical solution given the absence of datasets that contain both PCI records and detailed maintenance histories. To minimize potential bias, we aligned image selection with PCI assessment years and verified repairs at the same locations to ensure consistency.”

8. 162-166: Manual annotation procedure is not described in enough detail. How many? Was inter-rater agreement measured?

**Author response:** Thank you for pointing this out. We have expanded the description of the manual annotation process to provide additional detail. The revised text reads as follows:

“In addition, manual annotations were conducted by members of the research team to support the evaluation of the models’ identification capabilities. Each image was independently reviewed by annotators who answered a series of structured questions corresponding to the model prompts. A total of 29 questions were answered for each image. These questions focused on the presence and type of pavement defects, their spatial distribution, and relevant environmental or contextual conditions. Specifically, annotators assessed (1) surface features, such as whether cracks or potholes were present and, if so, the general type of cracking (transverse, longitudinal, or alligator); (2) spatial distribution patterns, indicating whether defects appeared isolated, spread across the pavement surface, or concentrated along joints, edges, or drainage areas; and (3) environmental and contextual conditions, including visible intersections, drainage features, patched or sealed areas, signs of resurfacing, and evidence of poor drainage or standing water that could exacerbate pavement deterioration. In cases where annotators disagreed, the discrepancies were discussed collectively until a consensus was reached.”

9. 205-208: definition of appears very subjective. any formal coding protocol or double-checking used?

**Author response:** Thank you for pointing this out. We have revised the definition of hallucination and clarified how it was identified in our study. The revised text now reads:

“Hallucination refers to cases where the model generates outputs that are unsupported, incorrect, or irrelevant based on the input image and prompt. In this study, if the described feature or condition could not be identified from the image, the response was classified as a hallucination.”

10. Statistical testing is ok, but effect sizes should also be reported to quantify practical differences, not just p values

**Author response:** Thank you for this helpful suggestion. We have conducted additional analyses and revised the manuscript accordingly to include effect sizes alongside the p-values. The following paragraph has been added to the section on ***Assessment dimensions***:

“Overall computational intensity and cost were used to evaluate efficiency. These were quantified by recording each model’s token usage and response generation time. Statistical significance in model differences was tested using the Kruskal–Wallis test, followed by pairwise Wilcoxon rank-sum tests with Bonferroni correction. To complement these tests, effect sizes were calculated to assess the magnitude and practical significance of the observed differences. For pairwise comparisons, rank-biserial correlation (*r*) was used, and for overall group differences, eta squared (η²) was computed based on the Kruskal–Wallis statistic. For proprietary models, token usage was further translated into estimated monetary cost based on publicly available pricing, enabling comparison of cost-efficiency.”

In addition, we have incorporated the effect size results into the section on ***Overall computational intensity and cost***, which now reads:

“A Kruskal-Wallis test revealed significant differences in token usage among the three models (p = 0.0066), with a small effect size (η² = 0.010). Post hoc pairwise Wilcoxon rank-sum tests with Bonferroni correction showed that Gemini 2.5 Pro used a significantly different number of tokens compared to both GPT-4o (adjusted p = 0.027, r = 0.11) and OpenAI o1 (adjusted p = 0.018, r = 0.12), while no significant difference was found between GPT-4o and OpenAI o1 (adjusted p = 1.000, r = 0.04). Despite statistical significance, the small effect sizes suggest that these differences in token usage may have limited practical relevance on their own.”

Results:
11. GPT-4o and OpenAI o1 are highlighted as top-performing, ut GEmma 3 is under-discussed even though it reached 100% response in one task, Being open-source, might be more important for reproducibility and cost.

**Author response:** Thank you very much for this thoughtful comment. We agree that Gemma 3 requires further discussion given its strong performance and relevance for reproducibility and cost-efficiency. In response, we have expanded **both the *Results* and *Discussion, limitations and future work*** sections to highlight the performance of open-source models, particularly Gemma 3. In the ***Results*** section, we added:

“Compared to proprietary models, open-source models generally outperformed proprietary models in spatial pattern recognition. LLaVA v1.6 Mistral (59.24%), LLaVA v1.6 Vicuna (58.48%), and Gemma 3 (48.61%) achieved higher response rates than GPT-4o (28.98%) and OpenAI o1 (29.58%), suggesting that open-source architecture may be better suited for spatial reasoning tasks. Nevertheless, all models showed markedly lower response rates in spatial pattern recognition than in other tasks, underscoring the persistent difficulty of capturing spatial context and relational features in road surface imagery.”

In the ***Discussion, limitations and future work*** section, we further elaborated on Gemma 3’s strengths:

“Among the open-source models evaluated, Gemma 3 is recommended as the base model for fine-tuning. It demonstrates high response rates across all tasks, including a perfect score (100%) for maintenance interval estimation. Gemma 3 also achieves the highest F1 score among open-source models, exceedingly even that of Gemini 2.5 Pro, which indicates strong reliability and balanced performance. Furthermore, Gemma 3 shows higher consistency with proprietary models compared to other open-source models. In contrast, the relatively low consistency observed among the LLaVA and Llama models suggests that their responses may be more variable. Overall, Gemma 3 achieved the best performance across all tasks, making it a strong candidate for domain-specific fine-tuning and adaptation.”

12. Hallucination analysis would benefit from systematic quantification across tasks and models

**Author response:** We sincerely appreciate this thoughtful suggestion. In response, we have clarified the scope and purpose of the hallucination analysis in the revised manuscript, specifically in the section ***Multimodal errors***. As described in the section on ***multimodal errors***, hallucination patterns are now discussed in relation to specific tasks, illustrating where and how they occurred. A systematic quantification by model was not conducted because hallucinations did not consistently appear across all tasks, models, or individual runs, which makes uniform measurement statistically unreliable. Instead, the hallucination analysis was designed as a qualitative complement to the three quantitative indicators that already provide a comprehensive and systematic evaluation of model performance, including response correctness, pattern identification, and reasoning accuracy. This qualitative perspective helps to explain the contextual and reasoning-related causes of model errors and provides deeper insight into the quantitative findings.

13. the figures are difficult to read at this scale

**Author response:** Thank you for this helpful comment. We have revised all figures to improve readability in accordance with the journal’s formatting standards by setting the font size between 8 and 12 points (Arial) and ensuring a resolution of 300–600 dpi.

Limitations:
14. Actual pavement management requires more than just surface imagery?

**Author response:** Thank you for this insightful comment. We agree that effective pavement management requires more than surface imagery. To address this point, we have expanded the section ***The potential and current limitations of existing MLLMs in road surface condition assessment*** emphasize the broader data needs of pavement management and clarify the role of generative artificial intelligence (GAI). The revised text reads as follows:

“Finally, Although GAI can reduce reliance on manual inspections by automating visual assessments, human oversight remains necessary to validate cases and ensure alignment with engineering standards. While GAI using street-view imagery can assist with tasks related to surface condition assessment, effective pavement management requires additional data beyond surface imagery such as structural integrity and traffic loading. Therefore, GAI should be viewed as a decision-support toolkit that enhances human judgment rather than a complete replacement for professional expertise and field validation.”

15. Fine tuning and prompt engineering are mentioned but not fully discussed in domain specific needs

**Author response:** Thank you for this helpful comment. We have expanded the section ***The potential and current limitations of existing MLLMs in road surface condition assessment*** to provide a more detailed discussion on fine-tuning and prompt engineering in domain-specific applications. The revised text reads as follows:

“Another factor affecting model performance is the clarity and completeness of the input prompt. Models often produce inaccurate responses when the input lacks sufficient context. For instance, in this study, the prompt only asked the models to recommend maintenance intervals based on street-view images, without providing any additional background information. As a result, the models focused exclusively on visible pavement conditions and overlooked non-visual determinants of maintenance needs. Incorporating these contextual factors into model prompts or fine-tuning datasets could improve both the reliability and practical utility of MLLM-generated outputs.

Furthermore, generative models may produce probabilistic outputs, meaning that repeated runs do not necessarily yield identical results unless generation parameters are fixed. This stochastic variability parallels observer inconsistency in manual inspections and can introduce uncertainty into the evaluation process. Standardizing model parameters and employing well-structured prompts can mitigate this randomness, thereby enhancing the reproducibility and reliability of MLLM outputs.”

16. Comparison between Open source and proprietary could be expanded

**Author response:** Thank you for the helpful suggestion. We have expanded the comparison between open-source and proprietary models in the section ***Proprietary vs. open-source models: trade-offs and implications for future development***. The revised text reads as follows:

“This study compared the performance of proprietary and open-source MLLMs across multiple dimensions, including responsiveness, accuracy, internal agreement, and cost. While both categories of models demonstrate similar levels of responsiveness, proprietary models generally achieve higher accuracy and show stronger internal agreement, producing similar outputs when presented with the same or slightly modified prompts. These advantages make proprietary models highly reliable for operational deployment; however, their use involves financial costs and limited transparency. Most proprietary models cannot be fine-tuned or adapted for domain-specific applications, and their token-based pricing structures can substantially increase computational expenses, especially in large-scale analyses.

In contrast, open-source models are freely available and offer greater flexibility for customization and domain adaptation. Although they typically perform slightly worse than proprietary models in accuracy and consistency, their transparency, accessibility, and modifiability make them valuable for research and development. Open-source models can be fine-tuned on task-specific datasets, enabling iterative improvement without recurring costs. This balance between performance and accessibility suggests that open-source models are a practical foundation for future pavement assessment applications, particularly in settings where reproducibility and budget constraints are critical.

Among the open-source models evaluated, Gemma 3 is recommended as the base model for fine-tuning. It demonstrates high response rates across all tasks, including a perfect score (100%) for maintenance interval estimation. Gemma 3 also achieves the highest F1 score among all open-source models, exceedingly even that of Gemini 2.5 Pro, which indicates strong overall reliability and balanced performance. Furthermore, Gemma 3 shows higher consistency with proprietary models compared to other open-source models. In contrast, the relatively low consistency observed among the LLaVA and Llama models suggests that their responses may be more variable or randomly generated. Overall, Gemma 3 achieved the best performance across all tasks, suggesting it is a strong candidate for fine-tuning and domain-specific adaptation. For applications involving knowledge distillation, GPT-4o is the most suitable proprietary model. It achieves high accuracy and internal consistency, comparable to OpenAI o1, but at a lower operational cost.”
