# Supplementary material for: Multimodal generative AI for automated pavement condition assessment: Benchmarking model performance
Source: PLoS One. 2026 Feb 12;21(2):e0340380. doi: 10.1371/journal.pone.0340380 (PMC12900301; doi:10.1371/journal.pone.0340380)
Supplement: Attachment — Submitted filename: 20251217_Response to Reviewers.docx [file pone.0340380.s006.docx]

**Manuscript PONE-D-25-46997R1**

**Response to Editor and Reviewers**

**Additional Editor Comments**

I have received all the reviewers' reports and completed the evaluation of the revised manuscript. Reviewer #2 still has an outstanding item. Please address this. Thank you!

**Authors’ response:** Thank you very much for your time and for reviewing the revised manuscript. We have addressed the remaining comment from Reviewer #2. A detailed explanation of the revisions made in response to this comment is provided in our response to Reviewer #2 below.

**Reviewers' Comments to the Authors: Reviewer**

**Reviewer #2**

Many thanks to the authors for addressing my comments. I can see that the new version is well improved. All my concerns have been addressed. However, I recommend that the authors ensure that the claims added are adequately supported by appropriate references before the final version is published.

**Authors’ response:** We sincerely thank the reviewer for the positive assessment of the revised manuscript and for this helpful suggestion. In response to this helpful suggestion, we carefully reviewed the claims added in the previous revision and extended this review to the references supporting all claims throughout the manuscript to ensure appropriate scholarly support.

To further strengthen the academic rigor of the manuscript, we modified several references, particularly in cases where non–peer-reviewed sources were previously cited, by replacing them with appropriate peer-reviewed literature. When a claim was already supported by multiple peer-reviewed studies, the corresponding non–peer-reviewed reference was removed. When a claim relied primarily on a non–peer-reviewed source, we replaced it with an appropriate peer-reviewed citation. In cases where no suitable peer-reviewed reference could be identified to support a given claim, both the claim and the corresponding non–peer-reviewed reference were removed.

**References removed**

References 13, 27, 58, 59, 71, 73, 75, 76, and 80 were removed.

**References updated or replaced**

- Reference 25 was replaced with:
  Whang SE, Lee JG. *Data collection and quality challenges for deep learning*. Proceedings of the VLDB Endowment. 2020;13(12):3429–3432.
  This supports the claim: *“Acquiring such data is often challenging and time-consuming due to the need for extensive manual annotation.”*
- Reference 28 was replaced with:
  Bandi A, Adapa PV, Kuchi YE. *The power of generative AI: A review of requirements, models, input–output formats, evaluation metrics, and challenges*. Future Internet. 2023;15(8):260.
  This supports the claim: *“Unlike conventional machine learning models that require extensive training datasets and pre-defined rules, generative artificial intelligence (GAI) models can respond adaptively to a range of inputs and generate task-relevant outputs with limited contextual information”*
- Reference 29 was replaced with:
  Lu K, Zhao X, Li M, Wang Z, Zhou Y, Wang J. *StreetSenser: a novel approach to sensing street view via a fine-tuned multimodal large language model*. International Journal of Geographical Information Science. 2025.
  This supports the claim: *“Leveraging these capabilities, recent studies have begun to apply MLLMs in urban research, particularly using street-view imagery to analyze built environments, urban form, and neighborhood conditions.”*
- Reference 30 was replaced with:
  Blečić I, Saiu V, Trunfio GA. *Enhancing urban walkability assessment with multimodal large language models*. In: International Conference on Computational Science and Its Applications. Springer; 2024.
  This further supports the same claim as reference 29.
- Reference 57 was replaced with:
  Zhang S, Mu H, Liu T. *Improving accuracy and generalizability via multimodal large language model collaboration*. Proceedings of the IJCNN. 2024.
  This supports the claim: *“When baseline performance is insufficient, these models can be fine-tuned to improve task-specific accuracy.”*
- References 68 and 69 were replaced with:
  Wu J, Zhong M, Xing S, Lai Z, Liu Z, Chen Z, Wang W, Zhu X, Lu L, Lu T, Luo P. *VisionLLM v2: An end-to-end generalist multimodal large language model for hundreds of vision–language tasks*. Advances in Neural Information Processing Systems. 2024.
  This supports the claim: *“In contrast, MLLM can address multiple tasks within a unified framework by combining visual perception with linguistic reasoning.”*
- Reference 74 was replaced with

Zhou, Q., Zhang, J., & Zhu, Z. (2025). Evaluating urban visual attractiveness perception using multimodal large language model and street view images. *Buildings*, *15*(16), 2970.

This supports the claim: *“Scholars have utilized street-view images to evaluate* *streetscape quality….”*

- Reference 96 was replaced with:
  Kuang J, Shen Y, Xie J, Luo H, Xu Z, Li R, Li Y, Cheng X, Lin X, Han Y. *Natural language understanding and inference with MLLMs in visual question answering: A survey*. ACM Computing Surveys. 2025.
  This supports the claim: *“First, they allow users to interact with the model using natural language prompts, removing the need for specialized programming expertise.”*
- Reference 98 was replaced with:
  Zhou X, He J, Ke Y, Zhu G, Gutiérrez-Basulto V, Pan J. *An empirical study on parameter-efficient fine-tuning for multimodal large language models*. Findings of ACL. 2024.
  This supports the claim: *“One key area is the need for additional domain-specific training data to support fine-tuning and improve model performance.”*
- Reference 99 has now been published as a peer-reviewed article, and the citation has been updated accordingly.
- Reference 100 was replaced with:
  Chen B, Zhang Z, Langrené N, Zhu S. *Unleashing the potential of prompt engineering for large language models*. Patterns. 2025.
  This supports the claim*: “At the same time, prompt engineering plays an important role in improving model outputs, particularly for context-dependent tasks.”*

In addition, to further enhance academic precision, we refined the wording of several claims while preserving their original meaning. For example, **Reference 56** was replaced with Wu J, Zhong M, Xing S, Lai Z, Liu Z, Chen Z, Wang W, Zhu X, Lu L, Lu T, Luo P. Visionllm v2: An end-to-end generalist multimodal large language model for hundreds of vision-language tasks. Advances in Neural Information Processing Systems. 2024 Dec 16;37:69925-75 *“Although MLLMs have not yet reached the point of full automation, they demonstrate versatility across tasks.”*

Finally, after updating the references, we slightly reorganized the relevant paragraph on page 6 to improve clarity and logical flow. The revised paragraph now reads:

“Recent advancements in GAI have opened new avenues for automating assessment processes across various domains (54, 55). Although MLLMs have not yet reached the point of full automation, they demonstrate versatility across tasks (56). Traditional machine learning approaches for surface-level road condition assessment are typically designed and trained for a single, well-defined objective such as defect detection (60-62), defect classification (63-65), or surface segmentation (66, 67). In contrast, MLLM can address multiple tasks within a unified framework by combining visual perception with linguistic reasoning (68, 69). In addition, when baseline performance is insufficient, these models can be fine-tuned to improve task-specific accuracy (57).”
